# National Partnership to Improve Dementia Care in Nursing Homes Campaign: State and Facility Strategies, Impact, and Antipsychotic Reduction Outcomes

**DOI:** 10.1093/geroni/igaa018

**Published:** 2020-06-02

**Authors:** Stephen Crystal, Olga F Jarrín, Marsha Rosenthal, Richard Hermida, Beth Angell

**Affiliations:** 1 Institute for Health, Health Care Policy, and Aging Research, Rutgers, The State University of New Jersey, New Brunswick; 2 School of Nursing, Rutgers, The State University of New Jersey, Newark; 3 School of Social Work, Virginia Commonwealth University, Richmond; 4 School of Social Work, Rutgers, The State University of New Jersey, New Brunswick

**Keywords:** Alzheimer’s disease and related dementias, Antipsychotics, Chemical restraints, Sedative-hypnotics

## Abstract

**Background and Objectives:**

Antipsychotic medications have been widely used in nursing homes to manage behavioral and psychological symptoms of dementia, despite significantly increased mortality risk. Use grew rapidly during the 2000s, reaching 23.9% of residents by 2011. A national campaign for safer dementia care in U.S. nursing homes was launched in 2012, with public reporting of quality measures, increased regulatory scrutiny, and accompanying state and facility initiatives. By the second quarter of 2019, use had declined by 40.1% to 14.3%. We assessed the impact of state and facility initiatives during the Campaign aimed at encouraging more-judicious prescribing of antipsychotic medications.

**Research Design and Methods:**

Our mixed-methods strategy integrated administrative and clinical data analyses with state and facility case studies.

**Results:**

Results suggest that substantial change in prescribing is achievable through sustained, data-informed quality improvement initiatives integrating educational and regulatory interventions, supported by public quality reporting. Adequate staffing, particularly of registered nurses, is key to support individualized management of symptoms through nonpharmacological strategies. Case study results suggest that state and facility initiatives during the campaign achieved considerable buy-in for the goal of more conservative prescribing, through a social process of normalization. Reporting and reduction of antipsychotic use was not followed by increases in sedative-hypnotic medication use. Rather, sedative-hypnotic use declined in tandem with antipsychotic reduction, suggesting that increased attention to prescribing patterns led to more cautious use of other risky psychotropic medications.

**Discussion and Implications:**

Quality improvement initiatives to change entrenched but problematic clinical practices face many barriers to success, including provider-level inertia; perceptions that alternatives are not available; and family and staff resistance. Nevertheless, systemic change is possible through concerted, collaborative efforts that touch prescribing practices at multiple points; integrate educational and regulatory influences; activate local and state champions for improvement; foster reputational influences through public reporting and benchmarking; and support a social process of normalization of preferred care processes as a best practice that is in the interest of patients.

**Translational Significance:**

The success of state and facility initiatives to reduce antipsychotic prescribing in the National Partnership to Improve Dementia Care in Nursing Homes was greatest where they deployed multimodal strategies that integrated voluntary and mandatory features; education for multiple actors in the medication use process and regulatory components; collegial initiatives to achieve provider buy-in; use of data and public reporting as a motivator; and “normalization” of best practices within the provider community.

Management of behavioral and psychological symptoms of dementia is a major quality issue in long-term care of older people. Symptoms such as agitation, aggression, crying, cursing, wandering, or threatening others can be highly distressing for staff, other residents, and families, often leading to requests for clinicians to “do something” about these behaviors. The response is often an antipsychotic prescription. However, for frail elderly residents, these medications bring substantial risk. Despite the risks, antipsychotics are widely used for nursing home residents with dementia, in the United States and internationally. The gold standard of care for managing symptoms of dementia utilizes behavioral management strategies and environmental modifications, requiring substantial investments in staffing and education (1).

In the United States, reducing antipsychotic use has been an ongoing policy challenge. The 1987 Nursing Home Reform Act (OBRA-87) sought to reduce both physical restraint and antipsychotic use, referred to as “chemical restraints.” Under OBRA-87, a federally directed, state-operated system of oversight was created (2). Components included a survey and certification process entailing periodic site visits by regulators, empowered to issue deficiency citations, and the Minimum Data Set (MDS) system under which facilities provide periodic information on resident characteristics, treatments, and services. MDS data provide the source for public reporting of quality measures at the state and facility level, with data on individual facilities available to the public through Center for Medicare and Medicaid Services’ (CMS) Nursing Home Compare data set. The existence, in the United States, of this national system of public reporting provides an important framework for the addition of new quality measures to address emerging health care challenges.

In 2008, the Food and Drug Administration imposed a black box warning of increased mortality risk on all antipsychotic medications for elderly patients with dementia that reads “Warning: Increased Mortality For Elderly Patients With Dementia Related Psychoses,” following earlier warnings on second-generation antipsychotics. The Food and Drug Administration has estimated that such treatment is associated with a 1.6–1.7 times greater risk of death compared to placebo, based on a meta-analysis of 17 double-blind, randomized, controlled trials averaging 8–12 weeks with a total of 5,106 patients (3). In these trials, about 4.5% of drug-treated patients versus 2.6% of placebo-treated patients died, implying about two more deaths per 100 antipsychotic-treated than placebo-treated patients. Other studies support similar estimates of substantially increased mortality through multiple pathways, including stroke, acute myocardial infarction, infections including pneumonia, and other causes (4–6).

Antipsychotic use declined following the enactment of OBRA-87 (7), but during the late 1990s and 2000s, use increased as second-generation antipsychotics, perceived as safer, replaced first-generation ones. Increasing evidence of mortality with second-generation antipsychotics, culminating in the Food and Drug Administration black box warnings, had only limited impact on prescribing patterns (8). By 2011 (fourth quarter), 23.9% of residents were receiving antipsychotic medications, excluding those with schizophrenia, Huntington’s disease, or Tourette’s syndrome (9). Persistently high use despite growing evidence of mortality led to calls for action (10,11).

Early in 2012, the CMS, state agencies, nursing homes, advocacy groups, and other stakeholders jointly launched the Partnership to Improve Dementia Care in Nursing Homes, with an initial goal of reducing antipsychotic medication use (8). The Partnership aims to enhance the quality of life for people with dementia, protect them from substandard care, and promote goal-directed, person-centered care for every nursing home resident. This is accomplished through a multidimensional approach that includes public reporting, state-based coalitions, research, training, and surveyor resources.

At the start of the National Partnership Campaign to Improve Dementia Care in Nursing Homes, CMS added public antipsychotic use reporting for long- and short-stay residents, separately, at both the facility and state levels. In order to reduce the potential for underreporting, the reporting requirement applied to all residents (with or without recorded dementia) with only limited exceptions for those diagnosed with schizophrenia, Tourette’s syndrome, or Huntington’s disease. This public reporting process proved to be an important tool and motivator both for facility- and state-level quality improvement as the campaign progressed.

To examine the change in prescribing during the Partnership campaign, we examined predictors and trends in MDS-reported antipsychotic prescribing. Given concerns that initiatives to reduce antipsychotic prescribing might lead to shifts to sedative-hypnotic medications, we also examined predictors and trends in the use of this class of medications (12).

## Antipsychotic Utilization Trends in CMS Data, 2011–2019

**Figure 1.**
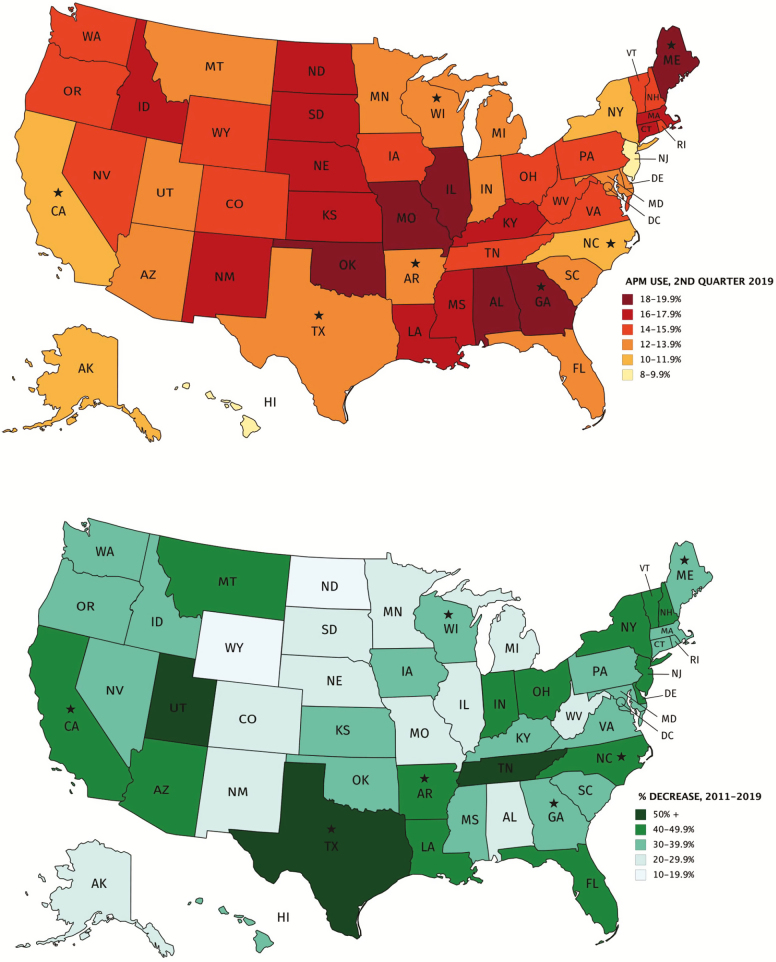
Antipsychotic prescribing (top) and change in antipsychotic prescribing (bottom) to long-stay nursing home residents by state, fourth quarter 2011 to second quarter 2019. States selected for key informant interviews are starred. *Notes*. Data from Center for Medicare and Medicaid Services (CMS) division of nursing homes, national partnership to improve dementia care in nursing homes: antipsychotic medication use data report (October 2019). Use rates are calculated by CMS from the minimum data set (MDS), version 3.0, and represent the proportion of long-stay residents without a diagnosis of schizophrenia, Huntington’s disease, or Tourette syndrome, who received an antipsychotic medication within the 7 days preceding the MDS assessment. Long-stay residents are defined by a total of 101 days or more without a gap of 30 contiguous days living in the community or other institution (14).

The most current data on antipsychotic use are provided in tables periodically compiled by the CMS Division of Nursing Homes (13). These data show a relative decrease of 40.1%, from 23.9% (fourth quarter 2011) to 14.3% (second quarter 2019). Nationally, reduction slowed somewhat after 2015 and plateaued in 2018. However, over the full 2011–2019 period, most states achieved significant improvement, with much of the national improvement driven by reductions in several large states, including Texas, New York, California, and Florida (13). Figure 1 presents: (top) state rates of use in 2019; and (bottom) change in use in each state from 2011 to 2019. The largest improvements were seen in Texas (−57.2%), Utah (−54.5%), Tennessee (−51.5%), Arkansas (−49.1%), New York (−48.7%), California (−48.5%), North Carolina (−47.6), New Jersey (−46.8%), Florida (−46.0%), Louisiana (−45.8%), New Hampshire (−45.3%), Arizona (−44.9%), Indiana (−42.2%), Vermont (−41.5%), and Delaware (−40.0%).

## Research Design and Methods

We triangulated data from three sources: (a) long-stay resident assessments linked with nursing home facility-level data; (b) nursing home facility case studies; and (c) state case studies.

### Resident and Facility Data

National MDS data available to the team (2011–2016) were linked with the Certification and Survey Provider Enhanced Reports (CASPER). This timeframe is congruent with the timeframe of the state and facility case studies and provides a focus on the period when new initiatives, reporting systems, and regulatory changes were being implemented by CMS and the National Partnership. MDS includes resident demographic information, clinical measures including behavioral symptoms of dementia, and antipsychotic and/or sedative-hypnotic medication administration at the time of assessment. CASPER includes nursing home characteristics such as ownership, number of beds, and proportion of Medicaid residents. We also used acuity-adjusted nurse staffing data from the CMS Nursing Home Compare database to adjust for the patient acuity or frailty of a facility’s residents. Our final sample included 21,431,330 assessments (quarterly observations) for 3,687,901 long-stay nursing home residents in 17,289 facilities during calendar years 2011–2016.

Using these linked data, we examined variation and change in MDS-reported antipsychotic and sedative-hypnotic prescribing, among residents without schizophrenia, Huntington’s disease, or Tourette’s syndrome. This method parallels the CMS Nursing Home Compare measure for inappropriate use of antipsychotic medication in nursing home residents. First, we examined variation in patient and facility characteristics between 2011 and 2016. Next, we used cross-sectional logistic regression models (2011 and 2016) to assess the pattern of the relationship between patient- and facility-level predictors and antipsychotic or sedative-hypnotic medication use. The decision was made to include bipolar disorder as a covariate, rather than exclusion, in light of Carnahan and Letuchy’s finding that among nursing home residents, bipolar disorder diagnosis is frequently nonspecific and follows a diagnosis of dementia, and to be generally consistent with the CMS measure (15). After determining the stability of the predictors over time, we focused on the effect of time (quarter-year intervals) in the final logistic models, highlighting the impact of the National Partnership on prescribing patterns after controlling for patient and facility characteristics.

### State Case Studies

For insight on strategies used in state campaigns, we conducted focus group interviews in 2016 with key informants from public health and government agencies (National Partnership coalition participants) in seven states: Arkansas, California, Georgia, Maine, North Carolina, Texas, and Wisconsin. These states were selected to ensure a balance of regional representation, population, and baseline antipsychotic use rates. States were selected to be heterogeneous with respect to the rate of antipsychotic reduction between 2012 and 2014. Interview questions focused on each state’s efforts to engage in the National Partnership, the strategies used, challenges encountered, and critical ingredients of success. The state case studies helped us understand what the states were doing during the study period (2011–2016). We also used information on state strategies compiled by the Partnership (8).

### Facility Case Studies

To better understand decision making and change in prescribing, we conducted 40 semistructured interviews at 14 nursing homes in the same seven states where the case studies were completed. Two nursing homes were selected in each state. In total, we interviewed 30 nursing home staff (primarily Directors of Nursing, activities staff, social services staff, and nursing staff) and 10 prescribing physicians. Questions focused on the decision-making process related to the use of antipsychotic medication, effects of CMS regulation, barriers to change, and sources of improvement. Interviews were completed from January through June of 2017, helping us understand how facilities responded to their state initiatives described in the case studies.

## Results

Nationally, antipsychotic prescribing declined by 29% and sedative-hypnotic prescribing by 43% between 2011 and 2016 (Table 1). Reduction in antipsychotic medication use was particularly substantial for black (−36.2%) and Hispanic residents (−33.6%) compared to non-Hispanic white residents (−27.6%), highlighted in Table 1. Reduction in antipsychotic use was greater among residents without recorded behavioral symptoms of physical or verbal aggression than among those with these behaviors, suggesting a trend to more judicious use, more focused on residents with the most severe symptoms (16). Similarly, a reduction in antipsychotic use was less among residents with recorded bipolar disorder diagnoses than for other residents, and the proportion of residents with a recorded diagnosis of bipolar disorder increased from 3.3% in 2011 to 4.1% in 2016. The mean age of residents decreased slightly, from 80.2 years (*SD* 13.2) in 2011 to 79.6 years (*SD* 13.4) in 2016, and the proportion of male residents increased from 30.8% to 33.5%. The reduction in sedative-hypnotic use by more than one third in every demographic group during the same period suggests that initiatives to reduce antipsychotic use did not lead to a shift in prescribing more sedative-hypnotic medications, as some have feared.

**Table 1.** Changes in the Long-Stay Nursing Home Resident Population, Facility Characteristics, and Use of Antipsychotic or Sedative Medications between 2011 and 2016

	Population Characteristics		Antipsychotic Use			Sedative-Hypnotic Use		
	2011	2016	2011 (%)	2016 (%)	Relative Change (%)	2011 (%)	2016 (%)	Relative Change (%)
Resident-level variables	*n* = 1,116,476	*n* = 1,119,889	22.9	16.2	−29.3	6.3	3.6	−42.9
Male	30.8%	33.5%	24.8	17.4	−29.8	6.7	4.0	−40.3
Female	69.2%	66.5%	22.0	15.6	−29.1	6.1	3.4	−44.3
White	78.0%	75.9%	23.2	16.8	−27.6	6.5	3.7	−43.1
Black	13.1%	14.0%	21.0	13.4	−36.2	4.9	2.8	−42.9
Hispanic	4.7%	5.3%	25.9	17.2	−33.6	7.6	4.9	−35.5
Asian/other	4.2%	4.8%	19.6	13.2	−32.7	5.8	3.2	−44.8
Medicaid	68.7%	71.8%	22.8	16.4	−28.1	6.2	3.4	−45.2
Physical aggression	5.4%	4.1%	48.2	38.2	−20.7	4.3	2.5	−41.9
Verbal aggression	8.4%	7.0%	45.1	35.0	−22.4	6.0	3.5	−41.7
Bipolar disorder	3.3%	4.1%	66.6	60.7	−8.9	10.5	6.5	−38.1
Dementia	44.8%	46.1%	29.9	21.0	−29.8	4.4	2.4	−45.5
Anxiety	23.0%	29.5%	33.6	25.1	−25.3	9.9	5.5	−44.4
Depression	49.7%	50.5%	27.0	19.9	−26.3	7.3	4.3	−41.1
Facility-level variables	*n* = 15,530	*n* = 15,524						
For-profit	69.1%	69.6%	23.8	16.8	−29.4	6.8	3.9	−42.6
Government	6.6%	7.4%	22.3	16.8	−24.7	5.2	3.4	−34.6
Non-profit	24.3%	23.0%	20.3	14.3	−29.6	5.0	2.9	−42.0
Rural	25.1%	23.1%	23.3	17.2	−26.2	6.2	3.7	−40.3
Urban	74.9%	76.9%	22.7	15.9	−30.0	6.3	3.6	−42.9
High bed count	≥172 beds	≥170 beds	23.2	16.7	−28.0	5.8	3.3	−43.1
Low bed count	≤96 beds	≤95 beds	22.2	16.1	−27.5	6.0	3.5	−41.7
High Medicaid	≥77% Medicaid	≥76% Medicaid	26.7	19.7	−26.2	6.6	3.9	−40.9
Low Medicaid	≤55% Medicaid	≤53% Medicaid	19.3	13.6	−29.5	5.8	3.4	−41.4
Acuity-adjusted average RN min. per resident day								
Highest quartile	34 min	50 min	21.1	14.5	−31.3	5.1	3.0	−41.2
Second highest quartile	21 min	33 min	21.9	15.5	−29.7	5.8	3.3	−43.1
Second lowest quartile	17 min	26 min	23.1	16.3	−29.4	6.6	3.8	−42.4
Lowest quartile	12 min	17 min	25.4	18.3	−28.0	7.5	4.4	−41.3
Acuity-adjusted average LPN hours & min. per resident day								
Highest quartile	1.42 h	1.57 h	23.3	16.3	−30.0	6.9	4.1	−40.6
Second highest quartile	0.98 h	1.16 h	23.5	16.7	−28.9	6.6	3.8	−42.4
Second lowest quartile	0.87 h	0.93 h	22.8	16.0	−29.8	6.2	3.5	−43.5
Lowest quartile	0.55 h	0.57 h	21.8	15.6	−28.4	5.3	3.0	−43.4
Acuity-adjusted average CNA hours & min. per resident day								
Highest quartile	3.11 h	3.18 h	22.9	16.1	−29.7	6.8	3.8	−44.1
Second highest quartile	2.48 h	2.53 h	22.3	15.3	−31.4	6.1	3.7	−39.3
Second lowest quartile	2.17 h	2.22 h	22.6	15.9	−29.6	5.9	3.4	−42.4
Lowest quartile	1.79 h	1.83 h	23.6	17.3	−26.7	6.2	3.5	−43.5
Acuity-adjusted average total RN/LPN/CNA hours per resident day								
Highest quartile	4.40 h	4.90 h	22.5	15.7	−30.2	6.4	3.7	−42.2
Second highest quartile	3.62 h	4.01 h	22.4	15.3	−31.7	6.1	3.6	−41.0
Second lowest quartile	3.25 h	3.58 h	22.8	16.1	−29.4	6.3	3.5	−44.4
Lowest quartile	2.78 h	3.05 h	23.7	17.5	−26.2	6.3	3.6	−42.9

*Note*: Authors’ analysis of Minimum Data Set, Certification and Survey Provider Enhanced Reports (CASPER), and Nursing Home Compare (acuity-adjusted staffing) data, 2011 and 2016. Ownership (For-Profit, Government, Non-Profit), Rural, and Urban variables refer to the percentage of individuals within that classification, as opposed to the percentage of facilities with that classification. CNA = certified nurse assistant; LPN = licensed practical nurse; RN = registered nurse.

In the adjusted model (Table 2) for 2016, antipsychotic use was notably higher, as expected, for residents with physical aggression (odds ratio [OR] 2.10), verbal aggression (OR 2.04), or bipolar disorder (OR 8.52). Among facility characteristics, lower Registered Nurse (RN) staffing and higher Licensed Practical Nurse staffing were associated with greater risk of antipsychotic use. Certified Nursing Assistant staffing levels were not associated with risk of antipsychotic use. Both the resident’s individual Medicaid eligibility and the proportion of Medicaid residents in the facility were associated with greater odds of antipsychotic use. Risk of sedative/hypnotic use was greatest among residents of Hispanic ethnicity (OR 1.27), those who exhibited verbal aggression (OR 1.41), and those with symptoms of depression (OR 1.52). Like antipsychotic use, sedative/hypnotic use was correlated with lower RN staffing and higher Licensed Practical Nurse staffing. Higher Certified Nursing Assistant staffing was also associated with greater sedative/hypnotic use. While this may seem counterintuitive, it could reflect a consequence of the cost-saving practice of substituting less expensive Licensed Practical Nurses and Certified Nursing Assistants for RNs.

**Table 2.** Logistic Regression Models of Effects of Resident and Facility Characteristics on Receipt of Antipsychotic and Sedative-Hypnotic Medication

	Antipsychotic				Sedative-Hypnotic			
	2011		2016		2011		2016	
	OR, *p*	95% CI	OR, *p*	95% CI	OR, *p*	95% CI	OR, *p*	95% CI
Resident-level variables								
Male vs female	1.119***	1.106–1.132	1.082***	1.069–1.096	0.923***	0.905–0.940	0.943***	0.922–0.965
Age (10-y increase)	0.823***	0.819–0.826	0.796***	0.792–0.800	0.841***	0.835–0.846	0.827***	0.821–0.834
Black vs white	0.832***	0.818–0.846	0.737***	0.723–0.751	0.646***	0.627–0.665	0.666***	0.642–0.691
Hispanic vs white	1.084**	1.058–1.111	0.947***	0.923–0.972	1.066**	1.025–1.108	1.272***	1.219–1.329
Asian/other vs white	0.855***	0.832–0.880	0.859***	0.835–0.885	0.796***	0.760–0.834	0.912***	0.865–0.961
Medicaid insurance	1.000	0.989–1.012	1.052***	1.038–1.066	1.048***	1.028–1.068	0.847***	0.827–0.868
Physical aggression	2.067***	2.021–2.114	2.099***	2.046–2.152	0.864***	0.821–0.908	0.921*	0.859–0.989
Verbal aggression	2.114***	2.076–2.154	2.039***	1.998–2.081	1.040*	1.005–1.077	1.047	1.000–1.097
Bipolar disorder	7.504***	7.314–7.700	8.522***	8.337–8.712	1.356***	1.305–1.409	1.409***	1.350–1.470
Dementia	2.429***	2.402–2.457	2.302***	2.273–2.331	0.611***	0.599–0.623	0.571***	0.558–0.586
Anxiety	2.164***	2.135–2.193	1.819***	1.792–1.846	0.595***	0.579–0.612	0.586***	0.563–0.609
Depression	1.554***	1.537–1.571	1.591***	1.572–1.610	1.414***	1.390–1.439	1.558***	1.523–1.593
ADL score	0.990***	0.989–0.991	0.990***	0.989–0.991	0.961	0.959–0.962	0.964***	0.962–0.966
Facility-level variables								
For-profit ownership	1.111***	1.096–1.127	1.073***	1.057–1.090	1.209***	1.181–1.237	1.156***	1.123–1.191
Government ownership	0.983	0.960–1.007	1.038**	1.013–1.064	0.991	0.951–1.032	1.120***	1.067–1.175
Rural vs urban	1.063***	1.049–1.077	1.077***	1.061–1.092	0.943***	0.923–0.963	1.011	0.984–1.038
High vs low Medicaid	1.321***	1.299–1.342	1.224***	1.202–1.246	0.954**	0.929–0.979	0.927***	0.897–0.958
Lowest vs highest RN	1.132***	1.114–1.151	1.139***	1.119–1.160	1.331***	1.296–1.367	1.280***	1.238–1.324
Lowest vs highest LPN	0.936***	0.921–0.951	0.949***	0.933–0.966	0.823***	0.801–0.844	0.802***	0.776–0.829
Lowest vs highest aide	0.986	0.970–1.001	0.990	0.972–1.007	0.831***	0.810–0.852	0.915***	0.885–0.945
High vs low bed count	1.017*	1.001–1.034	1.014	0.997–1.032	0.981	0.956–1.008	0.953**	0.922–0.985

*Notes*: Minimum Data Set and Home Health Compare (acuity-adjusted staffing) data, 2011–2016. ADL = activities of daily living; CI = confidence interval; LPN = licensed practical nurse; OR = odds ratio; RN = registered nurse.

*p < .01, **p < .001, ***p < .0001.

The effect of the campaign to improve nursing home care of patients with dementia over time (first quarter of 2011 through fourth quarter of 2016) was assessed using models that controlled for individual, facility, and state variables (Table 3). By the end of 2016, the adjusted risk of antipsychotic medication use was nearly halved from the beginning of the campaign (OR 0.55) with even greater reduction in risk of sedative-hypnotic medication use (OR 0.44).

**Table 3.** Effect of Time on Antipsychotic and Sedative-Hypnotic Medication Use, Controlling for Individual, Facility, and State Variables

	Antipsychotic		Sedative-Hypnotic	
	OR, *p*	95% CI	OR, *p*	95% CI
2011 Q1	(ref)		(ref)	
2011 Q2	0.994	0.987–1.002	0.998	0.985–1.010
2011 Q3	0.994	0.986–1.002	1.004	0.992–1.017
2011 Q4	1.002	0.994–1.010	0.982*	0.969–0.995
2012 Q1	0.975***	0.967–0.983	1.013	1.000–1.026
2012 Q2	0.983***	0.976–0.991	0.952***	0.939–0.964
2012 Q3	0.959***	0.952–0.967	0.915***	0.903–0.927
2012 Q4	0.911***	0.904–0.918	0.874***	0.863–0.886
2013 Q1	0.861***	0.854–0.868	0.867***	0.856–0.878
2013 Q2	0.830***	0.824–0.837	0.820***	0.809–0.831
2013 Q3	0.803***	0.796–0.809	0.780***	0.770–0.790
2013 Q4	0.770***	0.764–0.777	0.736***	0.726–0.746
2014 Q1	0.746***	0.741–0.752	0.721***	0.712–0.731
2014 Q2	0.730***	0.724–0.735	0.695***	0.686–0.705
2014 Q3	0.718***	0.713–0.724	0.657***	0.649–0.666
2014 Q4	0.704***	0.698–0.709	0.629***	0.621–0.638
2015 Q1	0.694***	0.689–0.700	0.620***	0.612–0.629
2015 Q2	0.657***	0.652–0.663	0.587***	0.579–0.595
2015 Q3	0.628***	0.623–0.633	0.556***	0.548–0.564
2015 Q4	0.602***	0.597–0.607	0.528***	0.520–0.536
2016 Q1	0.597***	0.592–0.602	0.525***	0.518–0.533
2016 Q2	0.579***	0.574–0.583	0.497***	0.490–0.504
2016 Q3	0.569***	0.565–0.574	0.470***	0.462–0.477
2016 Q4	0.549***	0.544–0.553	0.440***	0.433–0.447

*Notes*: CI = confidence interval; OR = odds ratio. Total observations = 21,431,330.

*p < .01, **p < .001, ***p < .0001.

### Facility Case Studies

Several recurring themes in the case study data provide additional insight into decision making and change, putting the results in context. First, facility staff and prescribers generally appreciated the risks of antipsychotic use and supported the need to reduce use and to treat these medications as a last resort. However, most were not aware of the National Partnership campaign. Despite this, responses suggested considerable staff and clinician buy-in to the campaign’s overall aim of reducing reliance on antipsychotic use.

A second recurring theme was the importance of systematic use of data for quality improvement. For example, respondents reported on internal initiatives to analyze facility prescribing data and distribute results to the staff to support monitoring. A Director of Nursing reported: “We have a task force that’s working on reducing antipsychotics … we have a dashboard … we have the CASPER report. We run it monthly.”

Third, consistent with findings from a recent systematic review of decision making for dementia patients (17), respondents offered strong support for the essential role of collaboration and communication in safe dementia care practices. A recurring theme was that incorporating improved practices into prescribing and medication management processes across multiple levels of decision making required the efforts of interdisciplinary teams, including staff at all levels, particularly nursing assistants. Respondents also emphasized the importance of clear communication among staff and with physicians.

Fourth, respondents spoke about the challenge of and need for individualized approaches to behavioral issues. For example, a registered nurse noted:

a patient in the Alzheimer’s unit that kept urinating in the hallway on the floor, around the nurse’s cart … they tried redirection, they tried toileting, they tried all kinds of things … And then they have these lights that I bought at Wal-Mart that come on when you walk by, and I stuck it to the back of the bedside commode and he began to use the bedside commode instead of urinating in the hallway.

Fifth, to achieve such individualized approaches, respondents perceived a need for more training in the use of nonpharmacological strategies for symptom management. Nurses described in-service training and informal advice from other staff as useful but not sufficient to give nursing assistants, and even nurses, needed insight into the sources of dementia patients’ agitation and aggression, and methods for dealing with these behaviors: “ask every nurse in the facility, ‘Do you feel you’re getting the education you need to assist you when caring for these patients [with dementia]?’ Because I bet half of them would say, ‘No.’” Education on dementia management and the risks of pharmacological strategies was also reported to be important for family members. Some respondents observed that in their concern for an elderly relative’s well-being, and discouragement over aggressive or agitated behaviors, family members often see antipsychotics as a solution rather than a problem.

Finally, respondents were generally conscious of, and even supportive of, the changes in CMS regulations on antipsychotics, although some took a rather cautious view of monitoring by surveyors, sometimes seeing the surveyors as too focused on “the numbers” and not conscious of the complexities of reducing antipsychotic medication use. Several expressed concern that the CMS regulations and surveyors do not differentiate between antipsychotic medications prescribed for nursing home patients with dementia and those with severe mental illness. A physician commented, “[The Director of Nursing] does not want to take admissions for somebody that is on an antipsychotic agent because heaven forbid that will mess their numbers up … she is feeling pressure from the state surveyors and other people.”

Overall, the interviews suggest that reducing antipsychotic medications is more time- and resource-intensive than relying on medication, by requiring a person-centered approach. However, the consensus was that given appropriate staff time, training, and effective communication, individualized reduction of antipsychotic medications is achievable, as well as desirable.

### State Case Studies

State coalition respondents indicated the importance of multimodal strategies that involved both state-level interorganizational coordination and training and technical assistance at the facility level. In several states, respondents noted the important role, in sustaining these initiatives, of CMS grants from Civil Monetary Funds (funds derived from penalties paid by facilities for quality and safety violations). State respondents, like those in the facility studies, noted the importance of public reporting of antipsychotic use rates at facility and state levels, included on CMS’s Nursing Home Compare website beginning in July of 2012 (18). Public reporting served to define change targets and as a catalyst to action: one respondent from Georgia noted “the powerful motivator of shame.” As a California respondent stated, “When you compare people to a benchmark and to their peers and they’re not looking too good, that definitely gets their attention.”

Public reporting served as an incentive for improvement at both state and facility levels. Texas used metrics to identify facilities that achieved notable reductions in antipsychotic prescribing whose strategies could be shared with other facilities. Maine similarly identified high-improvement facilities and presented these data to state legislators and local media. Conversely, quality metrics were used to identify facilities in the greatest apparent need of support for quality improvement (termed by respondents “low-hanging fruit”). Texas identified the 100 facilities with the highest use of antipsychotics and sent certified letters to their Medical Directors, encouraging them to address the issue. Quality Improvement Organizations (QIOs) and regulators also used metrics to focus their efforts. QIOs assisted nursing homes to collect and interpret facility data over time to support monitoring efforts. These interventions were complemented by an increased regulatory focus on antipsychotic use during regular regulatory site visits (survey and certification process), in which each nursing home is visited periodically by a state survey team. In addition, special site visits focused on reviewing dementia care (focused dementia care surveys) were implemented during 2015 in Texas, California, and other states.

Once facility targets were identified, state coalitions developed or obtained training and technical assistance materials to redefine and normalize prescribing and psychosocial practices that rely on person-centered care principles to manage difficult behavior. These training strategies varied from home-grown pamphlets, to materials provided by CMS, to the purchase (using Civil Monetary Penalty funds) of consultation and materials on nonpharmacological strategies. States typically offered in-person training and created online repositories for ongoing access by facilities. For individual facilities identified as struggling to achieve improvement, QIOs and other coalition participants provided individual assistance, including on-site training, phone-based technical assistance, and facility-to-facility mentoring programs. In Texas, a designated Quality Monitoring Program (QMP), distinct from the survey process, worked with facilities identified as in need of improvement; technical assistance visits addressed monitoring procedures and staff training on evidence-based practices. To address family fears regarding resident behavior that could be a barrier to de-prescribing, some states developed educational materials for families that could be distributed by facilities.

State respondents also reported the importance of involving members of a variety of professional groups in coalition activities, including physicians and pharmacists. For example, in North Carolina, multiple coalition partners participated in training for facilities, including representatives of the state pharmacy association, medical directors, the ombudsman, the QIO, and CMS. Facility training addressed resources available to support improvement and detailed regulatory changes with which they would be expected to comply. Pharmacists in North Carolina were also highly involved in an effort to improve electronic medical records to allow facilities to easily and quickly identify resident-level information about antipsychotic medication use.

## Discussion and Implications

Several themes that influence antipsychotic medication prescribing in nursing homes emerged from this mixed-methods study. First, public reporting of a safe-use metric appears to have been a key element in motivating changes, at both state and facility levels. As a respondent from Texas noted, “I think that we all were disgusted with being in last place in the country. We were 51st for a long time.” Public reporting of metrics will likely be a useful tool to motivate further progress and respond to any backsliding.

Second, in the large and complex long-term care system, engagement of multiple stakeholders was vital. This process began at the national level, with leadership from CMS, the national nursing home associations, and other key stakeholders. At the state level, a diversity of organizations was engaged. States that achieved rapid success, such as North Carolina and Georgia, benefited from already-developed working relationships among CMS, the QIOs, statewide provider organizations, and individual facilities. These relationships were marshaled to develop new advisory groups to brainstorm strategies to assist facilities with high antipsychotic use. While these efforts were typically coordinated by the QIO, they benefited from established collaborations among key stakeholder groups. A North Carolina participant described high rates of attendance at initial rollout trainings in 2012 and explained that this pattern was typical in a state in which “facilities are very, very interested in being on the cutting edge of things.”

In the largest states, the extended time necessary to engage multiple geographically dispersed stakeholders and facilities emerged as an important theme. Trends in California, Texas, and New York earlier in the initiative (2012–2015) versus later (2016–2018) reflect the challenge of generating change in such large systems. The trajectory of change was slower in these states than smaller states; each achieved greater relative improvement later in the campaign, improving in rank relative to other states (Figure 1). These results suggest that achieving change in large state systems, with thousands of facilities, requires a sustained multiyear effort to engage the necessary range of stakeholders on a statewide basis. Once these initiatives are incorporated into these large systems, however, the experience of California, Texas, and New York suggests that sustained change can be achieved in such systems. However, continuing efforts will likely be required to institutionalize these changes. New initiatives may periodically need to be rolled out in order to maintain the energy brought to the field by state and federal oversight and educational campaigns, to maintain the attention of prescribers, facility staff, and other stakeholders. Recent plateauing in antipsychotic use rates suggests the challenges facing sustained and continuing improvement.

Third, integration of educational activities and regulatory oversight contributed to the effectiveness of state initiatives. Initiatives based on the survey and certification system, such as focused dementia care surveys in which antipsychotic prescribing was reviewed in detail (conducted in Texas, California, and other states during 2015), contributed to facilities’ motivation to incorporate improvement strategies into their operations. Respondents reported that regulatory feedback was most effective when it focused on improving internal review and quality management processes rather than individual cases. For example, one California respondent noted:

I’m not sure hitting people with a stick for pharmacological use would be as effective as forcing them to write a plan of a correction for care that is not meeting the standard of individualized dementia care including appropriate activities.

More broadly, and consistent with findings from systematic reviews of health system initiatives to change prescribing and other clinical practices (17,19,20), state-level initiatives appeared to be most successful when: (a) they achieved buy-in that the recommended practices were in the best interest of patients and (b) accomplished incorporation of desired practices into established workflows. A process of “normalization” of preferred practices, transmitted and reinforced through social processes among the stakeholders involved, helped to define reduction of antipsychotic medication as best practice, in the interest of patients and consistent with professional expectations. This process of normalization has been described in other contexts (21) and is a key feature of creating sustained changes in health care provider behavior (22). In the case of antipsychotic medication reduction in nursing homes, oversight and quality improvement initiatives were authoritative enough to engage prescribers, facility staff, and others involved in the medication decision-making process, while sufficiently collegial, evidence-based, and educationally oriented to achieve buy-in and normalization of preferred practices.

Fourth, facilities with the most severe understaffing appeared to have been less able to respond to incorporate the recommended practices into their care processes. While total nurse staffing is important, results suggest that substitution of lower-educated Licensed Practical Nurses and Certified Nursing Assistants for RNs may be problematic for this dimension of quality. In particular, lower registered nurse staffing was associated with greater reliance on antipsychotics. This finding is not surprising in view of the substantial differences in RN staffing reported across staffing quartiles. As given in Table 1, facilities in the lowest quartile (2016) averaged only 17 minutes of RN time per resident day, in contrast to 50 minutes for facilities in the highest quartile. Even in nursing homes staffed at the levels recommended by CMS, there may not be enough staff time for residents with behavioral symptoms of dementia to receive individualized activities and adequate physical activity during the day. Improving the infrastructure for recruiting and training nursing home volunteers (similar to requirements in the hospice industry) could help to improve personalized care for residents with dementia and lead to opportunities for volunteers to join the long-term care workforce (23).

Many quality initiatives to increase safety and quality in health care have had limited or no success. In contrast, the National Partnership has had substantial impact on a practice that has been widely considered a difficult target to change; that had persisted despite highly credible safety evidence; and that has been a challenge in many countries (24–27). What, then, was distinctive about this initiative that helped to drive its significant impact? Interventions varied across states, and the factors influencing prescribing across the nation’s nursing homes are complex.

### Policy Implications

Results suggest that the federally supervised, state-administered oversight structure for nursing homes created under OBRA-87 appears to have functioned well as a framework within which a campaign to address a specific problematic practice can operate effectively. In this regard, the success in reducing antipsychotic prescribing has similarities to earlier successful initiatives to reduce physical restraints, which, like antipsychotics, require a physician order, were emphasized in the regulatory/survey process as a target of improvement, and were publicly reported (28). The use of physical restraints declined from 41% in the early 1990s (29,30) to current rates of less than 3% (18). As with reducing antipsychotic use, reducing reliance on physical restraints required deployment of individualized strategies in managing patients with complex behavioral disturbances and communications challenges, as well as changing established mindsets concerning appropriate treatment practices.

Deploying alternative nonpharmacological strategies in place of medication-based strategies requires adequate RN staffing for individualized care planning and supervision of direct care staff. As reflected in all but the top quartile of nursing homes, current federal requirements do not assure staffing levels adequate to provide safe, individualized care. Stronger requirements and incentives to meet CMS minimum safe staffing guidelines would contribute to safer dementia care. The potential for substitution of pharmacological for psychosocial strategies for managing patients with dementia is heightened in the nursing home setting by misaligned financial incentives because, for long-term residents, facilities are responsible for staffing costs but not for the costs of medications, typically reimbursed by Medicare. This financial misalignment strengthens the argument for stronger federal staffing requirements and maximal transparency of staffing patterns.

Staffing adequacy is, of course, directly related to Medicaid reimbursement for long-term nursing home care, which varies widely across states and falls far short of Medicare reimbursement for postacute care provided in the same facilities. In consequence, facilities with the greatest dependence on Medicaid reimbursement are less able to provide the level of staff support necessary to design and implement personalized dementia care strategies that minimize reliance on antipsychotics. Although Medicaid-dominant facilities did achieve improvement, they remain more dependent on antipsychotic medications for symptom management. Given financial pressures on state budgets, the longstanding challenge of inadequate Medicaid nursing home rates is unlikely to be solved soon; however, current findings suggest the contribution of this challenge to patient safety problems.

Finally, continued financial and logistical support will likely be needed in order for state quality improvement consortia to sustain their efforts over time. CMS funding from Civil Monetary Penalty Funds, reported by some respondents as vital in their consortium’s success, will likely be needed on a sustained basis. As noted, continuing innovation with new educational and intervention strategies will be important, both to address the high level of turnover in facility staffs and clinicians and to provide novelty that continues to maintain stakeholders’ attention to these critical safety issues.

Overall, results suggest that safer dementia management, with reduced reliance on antipsychotics, is facilitated by approaches that effectively integrate educational and regulatory elements, public quality measure reporting, and adequate staff resources. Accelerated improvement several years into the campaign in several large states, relative to other states, suggests the importance of multiyear commitment to improvement initiatives in the larger systems. State and federal initiatives appear to have achieved considerable buy-in on the need to reduce antipsychotic use. Study results indicate that with a combination of educational and regulatory approaches, multi-stakeholder engagement, and measurement-based accountability, substantial improvement in safe dementia care in nursing homes is achievable. However, sustaining these efforts will require continuing collaborative effort. Adequate total nursing staffing and RN staffing, in particular, emerged as key factors, as facilities with lower staffing levels appeared to be less able to incorporate recommended changes. The importance of adequate staffing highlights financial concerns regarding the impact of reductions to state Medicaid programs and potential impact on voluntary efforts, including staffing above minimum levels.

The sustainability of the changes achieved by the campaign remains to be determined (31,32). Modifying practices in the large and complex long-term care system involves difficult challenges of changing established workflows and clinical habits. Thus far, the National Partnership campaign has demonstrated significant staying power and appears to have generated significant buy-in and incorporation of safer dementia care practices into established workflows. There appear to be grounds for optimism that if safer dementia care practices become embedded in ongoing care processes and in widely shared understandings of best practices, the National Partnership can achieve long-term impact. However, these efforts were challenged in 2020 by the COVID-19 pandemic, resulting in restrictions on visitors, volunteers, and group recreational and social activities that are a cornerstone of the National Partnership. At the same time, infectious disease epidemics such as COVID-19 highlight the importance of continued vigilance on antipsychotic prescribing, especially in periods where the quality of care is challenged by staffing shortages and other epidemic-associated difficulties (3–5,32).

Continued progress will likely require systematic continuing education for the large number of staff and physicians who flow through the long-term care system each year. Continued transparency of practices using public reporting of quality measures will also be important, along with integrated regulatory and educational initiatives to maintain focus on safe practices, and adequate staffing resources to provide personalized, patient-centered care.

Numerous online resources are available on the National Partnership to Improve Dementia Care website, including best practices to avoid unnecessary antipsychotic medication in nursing home residents living with dementia, and toolkits to promote sleep, reduce risk of infection, and reduce acute care transfers (8). Less is known regarding the outcomes of the focused dementia care survey process and its impact on the quality of care and life for residents in long-term care. To date, only pilot data are publicly available. Hopefully, the National Partnership Campaign to Improve Dementia Care will continue such efforts in their overall goal of creating environments that support person-centered care for individuals living with dementia.
